# Beweg-Gründe im Alter: Multivariate Analyse der Einflussfaktoren körperlicher Betätigung im Alter

**DOI:** 10.1007/s11553-022-00963-z

**Published:** 2022-07-05

**Authors:** Lukas Richter, Barbara Gösenbauer

**Affiliations:** 1 Department Soziales, Fachhochschule St. Pölten, St. Pölten, Österreich; 2 Institut für Gesundheitsmanagement, IMC Fachhochschule Krems, Krems an der Donau, Österreich

**Keywords:** Körperliche Aktivität, Österreich, Sozialgerontologie, Prävention, Bewegungsförderung, Physical activity, Austria, Social gerontology, Prevention, Physical activity enhancement

## Abstract

**Hintergrund:**

Körperliche Bewegung gilt als ein Schlüsselfaktor für die Gesundheitserhaltung im Alter, wobei die COVID-19-Pandemie („coronavirus disease 2019“), wie Studien zeigen, zu einer Reduktion der körperlichen Aktivität beigetragen hat und es nun Überlegungen bedarf, wie eine (Re‑)Aktivierung der älteren Bevölkerung gelingen kann. Ein Ansatz ist hierbei zu fragen, welche Faktoren körperliche Aktivität bereits vor der Pandemie positiv beeinflusst haben, um diese durch Interventionen gezielt anzusprechen.

**Fragestellung:**

Ziel der vorliegenden Untersuchung ist es, in einer multivariaten Analyse jene wichtigen Faktoren körperlicher Aktivität zu identifizieren, um so (Re‑)Aktivierungschancen aufzuzeigen.

**Material und Methode:**

Die hierfür durchgeführte Analyse mittels multipler logistischer Regression stützt sich auf Daten einer standardisierten, repräsentativen Telefonbefragung (*n* = 2042) der 60-jährigen und älteren Bevölkerung im österreichischen Bundesland Niederösterreich, die im Frühsommer 2019 durchgeführt wurde.

**Ergebnisse:**

Ein positiver Gesundheitszustand sowie motivationale Faktoren erhöhen die Chance auf regelmäßige körperliche Aktivität. Die soziale Komponente sowie die Überzeugung, dass Sport der Gesundheit guttut, steigern ebenfalls die Wahrscheinlichkeit, Bewegung zu machen. Soziodemografische Faktoren abseits des Einkommens konnten im Modell hingegen nicht als signifikante Prädiktoren identifiziert werden.

**Schlussfolgerungen:**

Um die (Re‑)Aktivierungschancen zu erhöhen, bedarf es Maßnahmen, welche die körperliche Aktivität als Mittel zu unterschiedlichen Zwecken älterer Menschen begreifen.

## Abstract (en)

**Background:**

Physical activity is a key factor for maintaining health in old age. Due to the coronavirus disease 2019 (COVID-19) pandemic, studies have shown that there has been a decrease in physical activity; thus, considerations are now needed on how to (re)activate older individuals. One approach is to ask which factors had a positive influence on physical activity before the pandemic and to address these through interventions.

**Objectives:**

The aim of the study is to identify important factors of physical activity in a multivariate analysis in order to show possibilities for (re)activation.

**Materials and methods:**

For this purpose, multiple logistic regression was conducted based on data from a standardized, representative telephone survey (*n* = 2042) of the 60-year-old and older individuals in the Austrian state of Lower Austria, collected in early summer 2019.

**Results:**

Positive health status and motivational factors increase the chance of physical activity. The social component and the perception of physical activity as good for one’s health also increases the likelihood of exercising. Sociodemographic factors, apart from income, could not be identified as significant predictors in the model.

**Conclusions:**

In order to increase the chances of (re-)activation, measures need to be taken that understand physical activity as a means to different ends for older people.

Vor dem Hintergrund der Gesundheitsförderung ist die körperliche Aktivität als ein zentraler Faktor zu nennen, welche jedoch aufgrund der COVID-19-Pandemie („coronavirus disease 2019“) von einem Teil der älteren Menschen eingeschränkt wurde. Entsprechend bedarf es Überlegungen, wie eine (Re‑)Aktivieren gelingen kann. Hierfür wirft der Beitrag mittels 2019 erhobener Daten einen Blick vor die Pandemie und identifiziert Faktoren, welche mit der körperlichen Aktivität assoziiert sind bzw. waren, um so Anschlussstellen einer (Re‑)Aktivierung aufzuzeigen.

## Hintergrund

Die evidenzbasierte Forschung hebt die positiven Effekte des Gesundheitsverhaltens für Gesundheit bzw. deren Erhalt im Alter hervor [5, 7, 20]. Als ein zentraler Faktor gilt hierbei die körperliche Bewegung, da in etwa 6 % aller weltweiten Todesfälle mit Bewegungsmangel assoziiert sind [26]. Bewegung vermindert nicht nur das Mortalitäts- und Erkrankungsrisiko, sondern hat präventive Effekte [11, 17], etwa hinsichtlich des Sturzrisikos [24], ebenso rehabilitative Effekte [23] sowie eine positive Wirkung auf das Wohlbefinden [15]. Körperlicher Aktivität und Bewegung ist dementsprechend eine umfassende salutogene Wirkung zuzusprechen [1, 10].

Trotz der Wichtigkeit erreichte die Mehrheit der älteren Menschen in Europa auch vor der Pandemie die WHO-Empfehlungen zur körperlichen Betätigung nicht [14] bzw. galt ein Drittel als inaktiv [9], wobei sich die Situation durch die Pandemie verschärft hat [25]. Soziale Distanzierungsmaßnahmen oder die Angst vor einer Ansteckung [8, 16, 19] trugen zu einer Reduktion der körperlichen Aktivität bei (wie bspw. Studien aus Niederösterreich zeigen [12, 13]), aufgrund derer mit einer Zunahme gesundheitlicher Beeinträchtigungen zu rechnen ist und es daher der wissenschaftlichen und gesundheitspolitischen Aufmerksamkeit bedarf [6, 21], wie eine (Re‑)Aktivierung erfolgen kann. Um sich dieser Frage zu nähern, ist eine Auseinandersetzung mit jenen Faktoren, welche das Aktivitätsniveau beeinflussen bzw. rahmen, nötig. Die vorliegende Arbeit hat dabei zum Ziel, auf Basis von vor der Pandemie erzeugten Befragungsdaten, in einem multivariaten Modell bereits im Fachdiskurs als wichtig bestimmte Faktoren in ihrem Einfluss auf die körperliche Aktivität der älteren Bevölkerung zu überprüfen und damit Anschlussmöglichkeiten für (Re‑)Aktivierungschancen aufzuzeigen.

Körperliche Aktivität ist entsprechend wissenschaftlicher Analysen mit sozioökonomischen, gesundheitlichen und psychosozialen Faktoren assoziiert [2, 3, 9, 14, 17, 18, 22] und folgende werden im vorliegenden Beitrag berücksichtigt: erstens der Gesundheitszustand, da nicht nur die körperliche Aktivität auf diesen wirkt [23, 24], sondern er eine Grundbedingung für die Möglichkeiten zur körperlichen Betätigung darstellt [2, 3, 9, 14]. Neben dem Gesundheitsempfinden wird dieser im Modell über das Hör‑, Seh- und Erinnerungsvermögen [9] abgebildet. Zweitens werden die allgemeine Orientierung an einer gesunden Lebensweise [2, 22] und die Selbstwirksamkeit [2, 3, 18] einbezogen. Drittens stehen Motive, wie beispielsweise die physische Aktivität mit dem Treffen von Freundinnen und Freunden zu verbinden und die Empfehlung zu Sport und Bewegung durch medizinisches Fachpersonal [2, 3, 17], mit körperlicher Aktivität im Zusammenhang und sind in die Untersuchung integriert. Viertens werden das Alter [9, 14, 17], das Geschlecht [18] sowie der Bildungsstand [14, 18] als signifikante Einflussgrößen in der Literatur genannt und berücksichtigt, wenngleich die Ergebnislage Inkonsistenzen aufweist [18]. Zudem steht die finanzielle Situation [14, 17] in einem positiven Zusammenhang mit der körperlichen Aktivität. Vor diesem Hintergrund wurden im Gesundheitsbarometer Alter NÖ 2019 entsprechende Variablen erhoben. Das vorliegende Modell berücksichtigt die genannten Faktoren und testet das Ausmaß ihrer Wirkung.

## Studiendesign

Die Analyse nutzt Daten des Gesundheitsbarometers Alter NÖ 2019. Hierbei handelt es sich um eine standardisierte, repräsentative Telefonbefragung im österreichischen Bundesland Niederösterreich, welche den Anspruch hat, empirische Daten zur gesundheitlichen Situation der 60-Jährigen und Älteren aus einer sozialgerontologischen Perspektive zu erheben. Das Sampling erfolgte nach Gemeindegrößen über eine vorab geschichtete Zufallsauswahl mit Screening nach dem Alter; die Stichprobe umfasst 2042 Personen. Aufgrund des Erhebungszeitraums von Juni–Juli 2019 bilden die Daten Bewegungsverhalten und -motive der älteren Bevölkerung vor der Pandemie ab. Die Analyse wurde mittels SPSS Version 27 (IBM Corp., Armonk, NY, USA) durchgeführt und die Ergebnisse basieren auf ungewichteten Daten.

Im binär logistischen Modell dient die Zustimmung der Befragten zur Aussage, regelmäßig Sport bzw. Bewegung zu machen, als abhängige Variable, die Antwortkategorien wurden in „trifft sehr oder ziemlich zu“ (1) bzw. „trifft wenig oder gar nicht zu“ (0) zusammengefasst (Tab. 1).

**Tab. 1:** Operationalisierung und Verteilung

Variable/Beschreibung		Kodierung	Verteilung im Datensatz (ungewichtet)
*Abhängige Variable*			
Regelmäßig Sport/Bewegung		0 = trifft wenig oder gar nicht zu	0 = 37 %
		1 = trifft sehr oder ziemlich zu	1 = 63 %
*Unabhängige Variablen*			
Gesundheitszustand	Gesundheitsempfinden	0 = negative Einschätzung	0 = 12 %
		1 = mittelmäßige Einschätzung	1 = 34 %
		2 = positive Einschätzung	2 = 54 %
	Hörvermögen	0 = eher schlecht 1 = eher gut	Hörvermögen: 1 = 72 %
	Sehvermögen (mit Sehhilfe)		Sehvermögen: 1 = 69 %
	Erinnerungsvermögen		Erinnerungsvermögen: 1 = 77 %
Orientierung	Achten auf eine gesunde Lebensweise	0 = selten bzw. gar nicht	0 = 24 %
		1 = sehr bzw. im Großen und Ganzen	1 = 76 %
Selbstwirksamkeit	Selbstwirksamkeit	1 (sehr niedrig) bis 13 (sehr hoch)	*M* = 8,597 (*SD* = 3,08)
Motive der sportlichen Betätigung	Empfehlung durch Arzt/Ärztin	0 = trifft nicht zu 1 = trifft zu	Ärztliche Empfehlung: 1 = 19 %
	Tut meiner Gesundheit gut		Gesundheit: 1 = 62 %
	Guter Zeitvertreib		Zeitvertreib: 1 = 18 %
	FreundInnen treffen		FreundInnen treffen: 1 = 17 %
	Ist mein Hobby		Hobby: 1 = 34 %
Soziodemografische Variablen	Alter	0 = 60–69	0 = 41 %
		1 = 70–79	1 = 38 %
		2 = 80+	2 = 21 %
	Geschlecht	0 = männlich	0 = 45 %
		1 = weiblich	1 = 55 %
	Bildung	0 = ISCED 5–8	0 = 19 %
		1 = ISCED 3–4	1 = 67 %
		2 = ISCED 1–2	2 = 14 %
	Einkommen	0 = unter 2000 €	0 = 42 %
		1 = 2000–unter 3000 €	1 = 31 %
		2 = 3000 € und mehr	2 = 27 %

*ISCED* International Standard Classification of Education

Die erklärenden Variablen können in fünf Kategorien – Gesundheitszustand, Orientierung, Selbstwirksamkeit, Motive der Bewegung und soziodemografische Faktoren – eingeteilt werden. Der *Gesundheitszustand* wird durch vier Indikatoren abgebildet: Das persönliche Gesundheitsempfinden, von „sehr gut“ (1) bis „sehr schlecht“ (5) bewertet, ist für die Analyse in eine *negative* („sehr schlecht und schlecht“), eine *mittelmäßige* („mittel“) und eine *positive* („gut und sehr gut“) Einschätzung zusammengefasst worden. Wie in Tab. 1 ersichtlich, gaben bspw. 12 % der Befragten eine negative Gesundheitseinschätzung an. Die Abfrage des Hör‑, Seh- und Erinnerungsvermögens erfolgte ebenso durch Selbsteinschätzung, wobei aufgrund der unausgeglichenen Antwortverteilungen diese in zwei Kategorien gebündelt wurden: eine „gute bzw. sehr gute“ Bewertung gilt im Modell als *eher gutes* (1) und ab einer „mittelmäßigen bis sehr schlechten“ Bewertung als* eher schlechtes *Vermögen. Zur allgemeinen Einschätzung der *gesundheitlichen Orientierung* wurden Daten der Frage genutzt, inwiefern die Befragten „gar nicht“ (4) bis „sehr“ (1) auf eine gesunde Lebensweise achten. Die Antworten wurden für das vorliegende Modell dichotomisiert. Fünf ausgewählte* Motive der sportlichen Betätigung* wurden den Befragten mittels Mehrfachantworten und den Antwortmöglichkeiten „trifft zu“ (1) bzw. „trifft nicht zu“ (0) vorgelegt. Die Selbstwirksamkeit wurde auf Basis von vier Items – diese lehnen sich an der General Self-Efficacy Scale an [4] – gemessen und ist metrisch skaliert. Als soziodemografische Variablen dienen das kalendarische Alter – metrisch abgefragt und in drei Kategorien gebündelt –, das Geschlecht, die Bildung nach ISCED-Klassifikation (International Standard Classification of Education) in drei Abstufungen und drei Einkommenskategorien.

## Ergebnisse

Die analysierte Stichprobe des logistischen Modells umfasst aufgrund fehlender Werte 1736 Beobachtungen. Mit einem Nagelkerke‑R^2^ von 0,594 sowie einem Hosmer-Lemeshow-Test von 0,394 bzw. einer ROC-AUC von 0,907 ist das statistische Modell als sehr gut zu bezeichnen. Die Odds Ratios (OR) sind in Tab. 2 dargestellt; entsprechend R^2^ können 59 % der Varianz erklärt werden.

**Tab. 2:** Logistische Regression – Einflussfaktoren auf die Chance körperlicher Aktivität

	Odds Ratio	95 %-KI		*p*
*Gesundheitsempfinden (Ref.: negativ)*				
Mittlere Einschätzung	1,426	0,864	2,354	0,165
Positive Einschätzung	**3,330**	1,913	5,795	0,000
*Hörvermögen (Ref.: eher schlecht)*	0,920	0,651	1,299	0,634
*Sehvermögen (Ref.: eher schlecht)*	**1,811**	1,312	2,499	0,000
*Erinnerungsvermögen (Ref.: eher schlecht)*	1,219	0,836	1,778	0,304
*Achten auf eine gesunde Lebensweise* *(Ref.: weniger/gar nicht)*	**3,449**	2,472	4,814	0,000
*Selbstwirksamkeit*	**1,181**	1,112	1,255	0,000
*Motive der Bewegung (Ref.: nein)*				
Ein Arzt/eine Ärztin hat es empfohlen	**1,411**	1,003	1,986	0,048
Tut der Gesundheit gut	**2,831**	2,111	3,797	0,000
Ist eine Möglichkeit, etwas mit FreundInnen zu unternehmen	**3,717**	2,228	6,203	0,000
Ist ein guter Zeitvertreib	**4,402**	2,807	6,903	0,000
Ist mein Hobby	**7,694**	5,398	10,968	0,000
*Alter (Ref.: 60–69)*				
70–79	0,950	0,685	1,317	0,758
80+	0,833	0,551	1,261	0,388
*Geschlecht (Ref.: männlich)*	1,267	0,946	1,696	0,113
*Bildung (Ref.: ISCED 5–8)*				
ISCED 3–4	1,011	0,665	1,538	0,959
ISCED 1–2	1,172	0,665	2,066	0,583
*Einkommen (Ref.: unter 2000* *€)*				
2000–unter 3000 €	**1,612**	1,155	2,249	0,005
3000 € und mehr	**2,275**	1,531	3,380	0,000
*χ*^*2*^*/Freiheitsgrade/p*	989,012/19/0,000			
*Nagelkerkes R*^*2*^	0,594			
*n (gültige)*	1736			
*Hosmer-Lemeshow χ*^*2*^*/p*	8,419/0,394			
*Fläche unter der ROC-Kurve*	0,907			

*ISCED* International Standard Classification of Education, *KI* Konfidenzintervall, *ROC* „receiver-operation-characteristics“

Die Indikatoren des Gesundheitszustands stehen mit der körperlichen Aktivität im Zusammenhang: Die älteren Befragten, die entlang des Gesundheitsempfindens zu einer positiven Einschätzung kommen, haben eine ca. 3‑mal so hohe Chance, regelmäßig Bewegung zu machen, wie Personen, die ihre Gesundheit als negativ einstufen (*OR* = 3,330). Unabhängig des Gesundheitsempfindens hat zudem das Sehvermögen (*OR* = 1,811) signifikanten Einfluss.

Nicht nur die Ausrichtung auf eine gesunde Lebensweise (*OR* = 3,449), sondern auch die Selbstwirksamkeit (*OR* = 1,181; steigt diese um eine Einheit, erhöht sich die Chance körperlich aktiv zu sein um das 1,18-Fache) sowie alle Motive tragen unabhängig voneinander zu einer Erhöhung der Chance, sich eher regelmäßig sportlich zu betätigen, bei. In Anbetracht der OR sind die Verankerung der sportlichen Betätigung als Hobby (*OR* = 7,694) und die Deutung als guter Zeitvertreib (*OR* = 4,402) besonders ausschlaggebende Faktoren. Hingegen stehen das kalendarische Alter, Geschlecht und Bildung in keinem signifikanten Zusammenhang mit Bewegung. Weitere Analysen mit hierarchischen Modellen (nicht abgebildet) zeigen, dass der zuerst signifikante Zusammenhang zwischen Alter und körperlicher Betätigung durch den Gesundheitszustand mediiert wird. Einzig das Einkommen erweist sich unter den sozioökonomischen Faktoren als signifikanter Prädiktor.

## Diskussion

Das Modell fasst die als wichtig identifizierten Einflussfaktoren für körperliche Betätigung im Alter zusammen und kann dabei nicht nur 59,4 % der Varianz erklären bzw. 83,5 % aller Fälle richtig klassifizieren, sondern die Prädiktoren in ihrer Bedeutung weitgehend bestätigen. Der erste Analyseblock verweist auf die Zentralität des Gesundheitszustands [2, 3, 9, 14], welcher als Wechselwirkung mit der körperlichen Betätigung gedacht werden muss: Dieser ist daher einmal mehr oder weniger Voraussetzung und zugleich Resultat körperlicher Aktivität. Wenig überraschend ist zudem der signifikante Einfluss des Sehvermögens, welches für viele sportliche/körperliche Aktivitäten nötig ist; gleichzeitig muss hier die Frage anschließen, wie sehbeeinträchtigte und blinde ältere Menschen in Bewegungsmaßnahmen einbezogen werden können. Entgegen vorangegangener Ergebnisse [9] ist die Erinnerungsfunktion in vorliegender Studie nicht signifikant. Die Inkonsistenz könnte jedoch auch ein Artefakt der Messung sein, die zitierte Studie [9] nutzt Daten aus einem Gedächtnistest und nicht – wie hier vorliegend – aus einem Selbsteinschätzer. Die Analyseblöcke zwei und drei machen auf die Zentralität intrinsischer und motivationaler Faktoren aufmerksam. Nicht nur können Ergebnisse bestätigt werden, welche körperliche Aktivität im Zusammenhang mit der Ausrichtung auf eine gesunde Lebensweise und Selbstwirksamkeit [2, 3, 18] konstatieren, sondern, dass die Chance auf sportliche Aktivität bzw. Bewegung beträchtlich steigt, wenn diese motivational begründet ist. Hierzu ist bemerkenswert, dass ärztliche Empfehlungen [2, 3, 17] und das Verständnis, dass körperliche Betätigung der Gesundheit gut tue, einen signifikanten und nicht vernachlässigbaren Einfluss haben, gleichwohl soziale Faktoren und die persönliche Integration in die Tagesstruktur (Zeitvertreib) bzw. Lebensweise (Hobby) eine noch einflussreichere Rolle einnehmen. Der soziodemografische Analyseblock verweist auf die Relevanz der finanziellen Situation [14, 17] und zeigt, dass geringe Einkommen wohl den Möglichkeitsraum für körperliche Aktivitäten verengen. Unter Kontrolle der anderen Faktoren sind Alter, Geschlecht und Bildung hingegen nicht signifikant, wie aber auch bereits andere Studien gezeigt haben [18]. Aus gerontologischer Sicht lässt sich argumentieren, dass es eben häufig nicht das kalendarische Alter an sich ist, sondern damit in Verbindung stehende Faktoren (wie der Gesundheitszustand) prädiktive Wirkungen entfalten; werden sie kontrolliert, geht der Zusammenhang mit dem Alter zurück oder verliert gar seine Signifikanz. Aus messtheoretischer Sicht muss auf die Abfrage der abhängigen Variable verwiesen werden: So stehen bestimmte Sport- und Bewegungsarten mal mehr oder weniger mit Geschlecht, Bildung und Alter in Zusammenhang [18]. In der vorliegenden Arbeit wurde aber allgemein nach Sport oder Bewegung gefragt und damit das Potenzial gezeigt, ältere Menschen für körperliche Bewegung anzusprechen.

### Limitation

In der vorliegenden Arbeit wurde auf die Daten einer telefonischen Befragung zurückgegriffen. Neben einem nicht untypischen Bildungsbias (mittlere und höhere formale Bildungsschichten sind etwas überrepräsentiert) ist aufgrund des Modus mit einer Unterrepräsentanz gesundheitlich schwerer beeinträchtigter älterer Menschen zu rechnen. Zudem ist anzumerken, dass die erfassten Daten auf Selbstangaben basieren, wodurch Verzerrungen nicht ausgeschlossen werden können. Weitere Variablen und die Möglichkeit longitudinaler Daten in die Pandemie hinein wären ein Zugewinn. Gleichwohl ist die Modellgüte als sehr gut zu bezeichnen und kann körperliche Aktivität zu einem hohen Grad erklären.

## Schlussfolgerungen

Wie gezeigt werden konnte, steht die körperliche Aktivität mit diversen Faktoren im Zusammenhang. Es gilt also Maßnahmen zu ergreifen, welche die körperliche Aktivität als Mittel zu unterschiedlichen Zwecken älterer Menschen begreifen. Gleichwohl die Bewegungsförderung im Kontext des Gesundheitserhalts zu denken ist, bedarf es also Maßnahmen, welche die soziale Komponente, d. h. die Kontakt- und Vergesellschaftungsfunktion, adressieren und körperliche Aktivitäten in den Tagesablauf und die Lebensweise integrieren. Bewegung bzw. Bewegungsangebote sind daher als Mittel der Alltagsgestaltung zu positionieren. Gleichzeitig müssen die Angebote älteren Menschen mit niedrigerem Einkommen und bereits schlechterem Gesundheitszustand offenstehen.

## Fazit für die Praxis

- Aus gerontologischer Perspektive ist nicht das kalendarische Alter, sondern der Gesundheitszustand für die körperliche Aktivität mitentscheidend. Mit steigendem Alter nimmt zwar die Wahrscheinlichkeit eines schlechteren Gesundheitszustandes zu, trotzdem sollten beide Faktoren in der Konzeption von Interventionen nicht gleichgesetzt werden.
- Insbesondere im Kontext von Altersarmut sind niederschwellige, d. h. kostengünstige bzw. kostenlose Bewegungsangebote zu forcieren, welche älteren Menschen durch körperliche Bewegung eine Plattform für Kontakte und Alltagsgestaltung bieten.
- Die (Re‑)Aktivierung der körperlichen Aktivität ist über gesundheitliche Aspekte hinaus zu denken und kann vor allem dann gelingen, wenn Maßnahmen in ihrer sozialen Funktion erkannt und im Tagesablauf älterer Menschen integriert werden können.
